# Helicobacter pylori Chronic Infection Selects for Effective Colonizers of Metaplastic Glands

**DOI:** 10.1128/mbio.03116-22

**Published:** 2023-01-04

**Authors:** V. P. O’Brien, L. K. Jackson, J. P. Frick, A. E. Rodriguez Martinez, D. S. Jones, C. D. Johnston, N. R. Salama

**Affiliations:** a Human Biology Division, Fred Hutchinson Cancer Center, Seattle, Washington, USA; b Molecular and Cellular Biology Graduate Program, University of Washington, Seattle, Washington, USA; c Department of Microbiology, University of Washington, Seattle, Washington, USA; d Vaccine and Infectious Disease Division, Fred Hutchinson Cancer Center, Seattle, Washington, USA; Emory University School of Medicine

**Keywords:** gastric intestinal metaplasia, genetic variation, *Helicobacter pylori*, outer membrane proteins, stomach

## Abstract

Chronic gastric infection with Helicobacter pylori can lead to progressive tissue changes that culminate in cancer, but how H. pylori adapts to the changing tissue environment during disease development is not fully understood. In a transgenic mouse gastric metaplasia model, we found that strains from unrelated individuals differed in their ability to infect the stomach, to colonize metaplastic glands, and to alter the expression of the metaplasia-associated protein TFF3. H. pylori isolates from different stages of disease from a single individual had differential ability to colonize healthy and metaplastic gastric glands. Exposure to the metaplastic environment selected for high gastric colonization by one of these strains. Complete genome sequencing revealed a unique alteration in the frequency of a variant allele of the putative adhesin *sabB*, arising from a recombination event with the related sialic acid binding adhesin (SabA) gene. Mutation of *sabB* in multiple H. pylori strain backgrounds strongly reduced adherence to both normal and metaplastic gastric tissue, and highly attenuated stomach colonization in mice. Thus, the changing gastric environment during disease development promotes bacterial adhesin gene variation associated with enhanced gastric colonization.

## INTRODUCTION

Helicobacter pylori stomach infection is the leading risk factor for the development of gastric malignancies, with an estimated 75% of gastric cancer cases attributed to active infection (1). Although the mechanisms by which H. pylori induces gastric cancer are incompletely understood, the tissue changes preceding cancer development have been well described (2). All infections lead to chronic inflammation of the stomach lining (gastritis). A fraction of these cases progress to atrophic gastritis (loss of acid secreting parietal cells), SPEM (spasmolytic polypeptide-expressing metaplasia) and/or IM (intestinal metaplasia), which then can progress to dysplasia and finally gastric cancer (3, 4). Eradication of H. pylori prior to the development of metaplasia reduces the risk of gastric cancer development (5), which supports a model in which H. pylori promotes early tissue alterations leading to carcinogenesis.

The interactions between host and pathogen are dynamic throughout infection as acid homeostasis, nutrient availability, and immune responses fluctuate (6). Infections are typically established in the stomach antrum, where pH is closer to neutral. However, loss of acid-secreting parietal cells due to atrophy of the stomach corpus glands promotes expansion of H. pylori’s niche from the antrum to the corpus in both human and animal models (7, 8). Within these different topographic regions, H. pylori can be found in the protective mucus layer above the epithelium and within glands (9–11). At the epithelial interface, H. pylori secretes effectors directly into host cells as well as into the surrounding environment, resulting in a robust inflammatory response (12). It is thought that by-products of inflammation generated from infection lead to accumulation of mutations in gastric epithelial cells, which are sufficient to activate oncogenic pathways in some individuals (13).

We recently showed that H. pylori presence alters the trajectory of SPEM, IM, and dysplasia (14), in addition to its previously described role in driving inflammation associated with oncogenic mutations. These studies suggest that H. pylori modulates premalignant tissue changes, which in turn can impose selective pressures on the infecting bacterial populations. H. pylori persistence in response to tissue remodeling in the gastric environment is facilitated by genomic diversification through mutation as well as inter and intra genomic recombination (15). Comparisons of paired H. pylori isolates collected longitudinally from the same individual document extensive genetic diversity, suggesting adaptation to distinct niches within a single host (16–18). However, few studies have characterized how H. pylori specifically interacts with, and may genetically adapt to, the metaplastic tissue environment. Here, we utilized a mouse model of KRAS-driven metaplasia to explore bacterial genotypes that promote colonization of the metaplastic stomach.

## RESULTS

### The model H. pylori strain PMSS1 robustly colonizes the gastric corpus during gastric preneoplastic progression.

**FIG 1:**
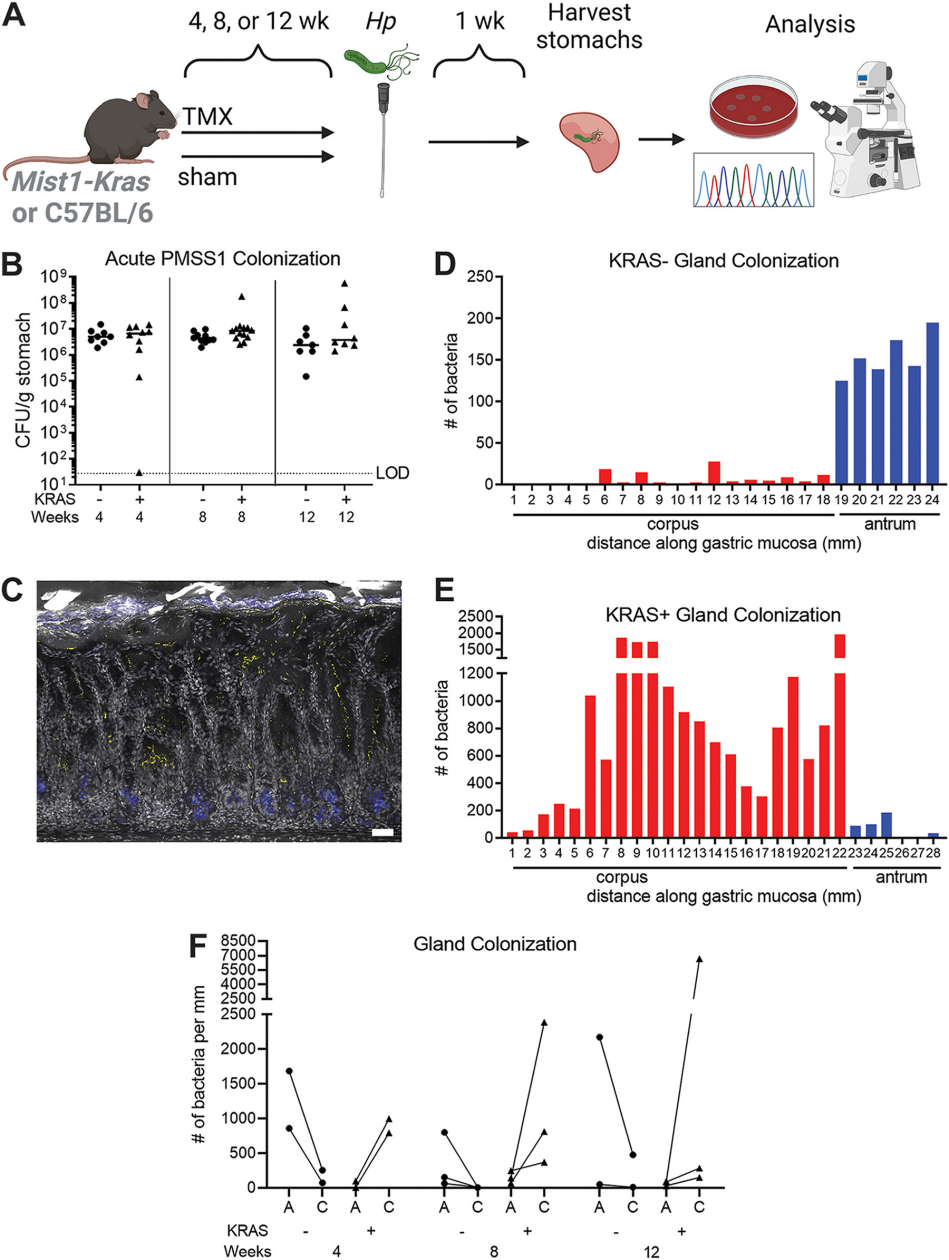
H. pylori colonizes the stomach corpus during gastric preneoplastic progression. (A) *Mist1-Kras* mice were given tamoxifen (TMX) to induce constitutively active KRAS in the chief cells, which leads to metaplasia development, or were sham-induced with vehicle (corn oil). After the onset of SPEM (4 weeks post-TMX), SPEM with intestinalizing characteristics (8 weeks), or intestinal metaplasia (12 weeks), mice were infected with Helicobacter pylori for 1 week to assess acute H. pylori-tissue interactions. Illustration created with BioRender.com. (B) At the indicated weeks after TMX treatment (+) or sham injection (−), mice were infected with PMSS1 for 1 week. Bacterial titers of individual mice are shown, and a bar indicates the median. Data are from N = 2 independent experiments with *n* = 7–13 mice per group. CFU, colony forming units; LOD, limit of detection. (C–E) Thick stomach sections (200 μm) were stained for H. pylori and imaged via confocal microscopy. Z-stacks were collected along the entire length of the stomach and volumetric analysis was used to enumerate bacteria based on fluorescent voxels. (C) Representative maximum intensity projection of a stomach section from the corpus of a mouse infected with H. pylori strain PMSS1 for 1 week, 12 weeks after KRAS induction. Gray, DAPI; yellow, *Hp*; blue, GS-II (metaplasia marker). Scale bar, 50 μm. (D–E) Shown are representative examples of gland analysis for PMSS1 in KRAS− (D) and KRAS+ (E) mice infected 8 weeks after induction or sham induction. The graphs show the number of bacteria within glands as detected by fluorescent voxels, along the length of the stomach in millimeters (mm). Red bars indicate the corpus and blue bars indicate the antrum. The range of the *y* axis differs between panels D and E. (F) Bacteria were enumerated in thick stomach sections from two to three mice per treatment and time point depicted in panel 1B. Each dot represents an individual mouse from panel 1B and lines connect values for the antrum (‘A’) and corpus (‘C’) from a given mouse.

We previously used *Mist1-Kras* mice to explore how H. pylori infection could impact metaplasia development in the stomach corpus (14). In these mice, administration of tamoxifen (TMX) induces expression of a constitutively active *Kras* allele (G12D) in chief cells, leading to rapid onset of spasmolytic polypeptide-expressing metaplasia (SPEM) in the corpus within 4 weeks, which progresses to intestinal metaplasia (IM) and mild dysplasia by 12 weeks (19). In the present study, we either induced active KRAS (KRAS+), or sham-induced mice (KRAS−), and then performed acute H. pylori infections to assess how H. pylori interacted with metaplastic versus healthy glands (Fig. 1A). We first tested H. pylori strain PMSS1, which robustly colonizes the healthy mouse stomach (8, 20). Mice were infected for 1 week during SPEM (4 weeks after KRAS induction), SPEM with intestinalizing characteristics (SPEM-IC, 8 weeks after KRAS induction), or IM (12 weeks after KRAS induction), with sham-induced mice as a control at each time point (Fig. 1B). PMSS1 robustly colonized the KRAS− mice at each time point, in keeping with its previously established ability to colonize wild-type mice. This strain also robustly colonized KRAS+ mice at each time point, demonstrating that it could survive in the metaplastic stomach. Interestingly, at 12 weeks, some KRAS+ mice had bacterial loads one to two logs higher than sham-induced mice. We hypothesized that this could result from H. pylori expansion from the antrum into corpus metaplastic glands.

In humans, H. pylori infection initially localizes to the antrum, which lacks acid-producing parietal cells, and then gradually can spread to the corpus (main body of the stomach) over a period of years to decades (21). A similar phenomenon was observed in mice: PMSS1 localized to the antrum after 1 week of infection in wild-type C57BL/6 mice and then spread to the corpus within 1 month (8). To visualize bacteria within glands, we used immunofluorescence microscopy to detect PMSS1 in thick sections (200 μm) of formalin-fixed tissue (Fig. 1C, Movie S1). We previously used immunofluorescence microscopy to detect and quantify H. pylori within gastric glands based on fluorescent voxels (8). Here, we used this method to compare antral and corpus gland occupation between KRAS− and KRAS+ mice infected with PMSS1. We examined stomach sections from two to three mice per time point. At each time point, more H. pylori cells were present within the glands of KRAS+ mice than KRAS− mice (Fig. 1D to F; note that the range of the *y* axis differs between 1D and 1E), suggesting that the metaplastic changes to the gland architecture and microenvironment may favor H. pylori gland colonization. Consistent with our hypothesis, far more bacteria were detected in corpus glands than antral glands of KRAS+ mice at each time point (Fig. 1F), whereas KRAS− mice maintained the expected antral predominance. Thus, H. pylori strain PMSS1 can robustly colonize metaplastic corpus glands.

10.1128/mbio.03116-22.1 MOVIE S1 Shown is a Z-stack of a mouse stomach infected with H. pylori strain PMSS1 (green) for one week, 12 weeks after tamoxifen was given to induce active KRAS. DAPI, blue, depicts nuclei. GS-II, red, labels metaplastic cells at the base of the glands. The tissue was imaged at 100× magnification on a Zeiss LSM 780 confocal microscope. Download Movie S1, MOV file, 4.7 MB. Copyright © 2023 O’Brien et al. 2023 O’Brien et al. https://creativecommons.org/licenses/by/4.0/ This content is distributed under the terms of the Creative Commons Attribution 4.0 International license.

### Genetically diverse H. pylori strains colonize the metaplastic stomach environment.

**FIG 2:**
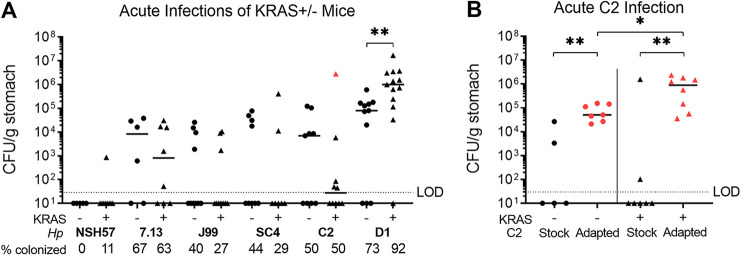
The recent isolate C2 can adapt to better colonize the mouse stomach. *Mist1-Kras* mice were sham-induced (−) or induced with TMX (+). After 8 weeks, mice were infected with H. pylori for 1 week. (A) Shown are stomach titers after 1 week of infection. The percentage of total mice colonized above limit of detection (10^1^ CFU/g of stomach tissue) is listed below each isolate name. The mouse from which the C2 “adapted” strain was isolated is indicated in red and used in additional experiments (B) in comparison to infection with the same C2-Stock culture (“stock”). Data points represent actual values from each individual mouse, bars represent the median, and mice with no detectable CFU are plotted below the limit of detection (LOD). Data are from N = 2–3 independent experiments. *, *P < *0.05; **, *P < *0.01, Mann-Whitney U test.

We tested a panel of H. pylori isolates to look for strains that might differ in their ability to colonize the metaplastic versus healthy stomach (Fig. 2A). NSH57 is a mouse-adapted version of the well-studied clinical isolate G27 but is a relatively poor colonizer of mice (22, 23). This strain colonized 0/5 KRAS− mice and 1/9 KRAS+ mice in our study. Strain 7.13 is a gerbil-adapted carcinogenic H. pylori strain derived from the duodenal ulcer strain B128 (24). Titers of 7.13 did not differ between KRAS− and KRAS+ mice. However, only 4/7 KRAS− mice and 5/8 KRAS+ mice were colonized, and median titers were three to four logs lower than titers of PMSS1, demonstrating that 7.13 is overall not a robust colonizer of mice, regardless of gastric metaplasia status. Thus, induction of metaplasia does not enhance stomach colonization by these strains.

We next tested infection with representative isolates from 4 distinct genetic subgroups within the J99 strain collection of H. pylori isolates taken from a single human host (25, 26). Based on shared genetic variation, the J99 isolates cluster in four distinct subgroups (27). Subgroups 1A and 1B represent single colony isolates collected from an antral biopsy specimen obtained in 1994, when the patient first presented with a duodenal ulcer. The patient refused to take the prescribed antibiotics to eradicate the infection. Subgroups 2A and 2B single colony isolates come from biopsy specimens taken 6 years later from the antrum, corpus and duodenum, when the patient progressed to atrophic gastritis (a precursor to metaplasia) with loss of acid secretion (28). The strains J99, SC4, D1, and C2 are representative of subgroups 1A, 1B, 2A and 2B, respectively.

The J99 strain was a relatively poor colonizer of mice, with titers recovered in only 4/10 KRAS− mice and 3/11 KRAS+ mice (Fig. 2A). The other strain from 1994, SC4, was likewise recovered from only 4/9 KRAS− mice and 2/7 KRAS+ mice. Strain D1 was recovered from 8/11 KRAS− mice and 12/13 KRAS+ mice, demonstrating that it could robustly colonize mice regardless of metaplasia status. D1 titers were about one log higher in KRAS+ mice than in KRAS− mice (*P < *0.01, Mann-Whitney U test), suggesting that this strain preferentially colonized the metaplastic stomach. The other strain tested from the later biopsy specimens, C2, was recovered from 5/10 KRAS− mice and 5/10 KRAS+ mice. We observed that the C2 strain was the most variable in overall bacterial burdens, with titers ranging from undetectable to greater than 10^6^ CFU/g stomach. We thus hypothesized that C2 might be poised to adapt to the metaplastic stomach environment.

To test this hypothesis, mice were infected with the C2 “stock” strain (as in Fig. 2A) or a C2 “adapted” strain recovered from a KRAS+ mouse, shown in red in Fig. 2A. Strikingly, titers of the C2-Adapted strain were significantly higher than the C2-Stock strain, regardless of KRAS expression (Fig. 2B). The C2-Stock strain was recovered from 2/5 KRAS− mice and 2/7 KRAS+ mice, whereas the adapted strain was recovered from all mice tested. Thus, C2 appears poised to adapt *in vivo* to efficiently colonize the mouse stomach. Titers of C2-Adapated were significantly higher in KRAS+ mice than KRAS− mice (median CFU 5 ×10^4^ versus 9 ×10^5^, respectively, Fig. 2B), suggesting that this adaptation may even favor the metaplastic environment. In these experiments we cultured bacteria from a portion of the stomach containing both the corpus and antrum. To explore whether there was a preferential niche for C2-Adapted, we cultured the corpus and antrum separately. All the detectable CFU were found in the corpus (Fig. S1).

10.1128/mbio.03116-22.2 FIG S1 C2-Adapted primarily colonizes the corpus. C57BL/6 mice were infected with C2-Adapted. At one week, mice were euthanized, and stomachs were aseptically harvested and opened along the lesser curvature. Stomachs were bisected longitudinally. One half, containing corpus and antrum (‘C+A’), was homogenized and H. pylori was cultured. The other half was cut again to separate corpus (‘C’) from antrum (‘A’) based on gross histology, and the pieces were homogenized for H. pylori culture. The results are plotted as colony-forming units (CFU) per gram of stomach tissue. Each dot represents an individual mouse and lines connect the values for the three pieces of tissue from a given mouse. Zeroes are plotted below the limit of detection (‘LOD’). Download FIG S1, TIF file, 0.1 MB. Copyright © 2023 O’Brien et al. 2023 O’Brien et al. https://creativecommons.org/licenses/by/4.0/ This content is distributed under the terms of the Creative Commons Attribution 4.0 International license.

### H. pylori isolates vary in their ability to infect metaplastic glands.

**FIG 3:**
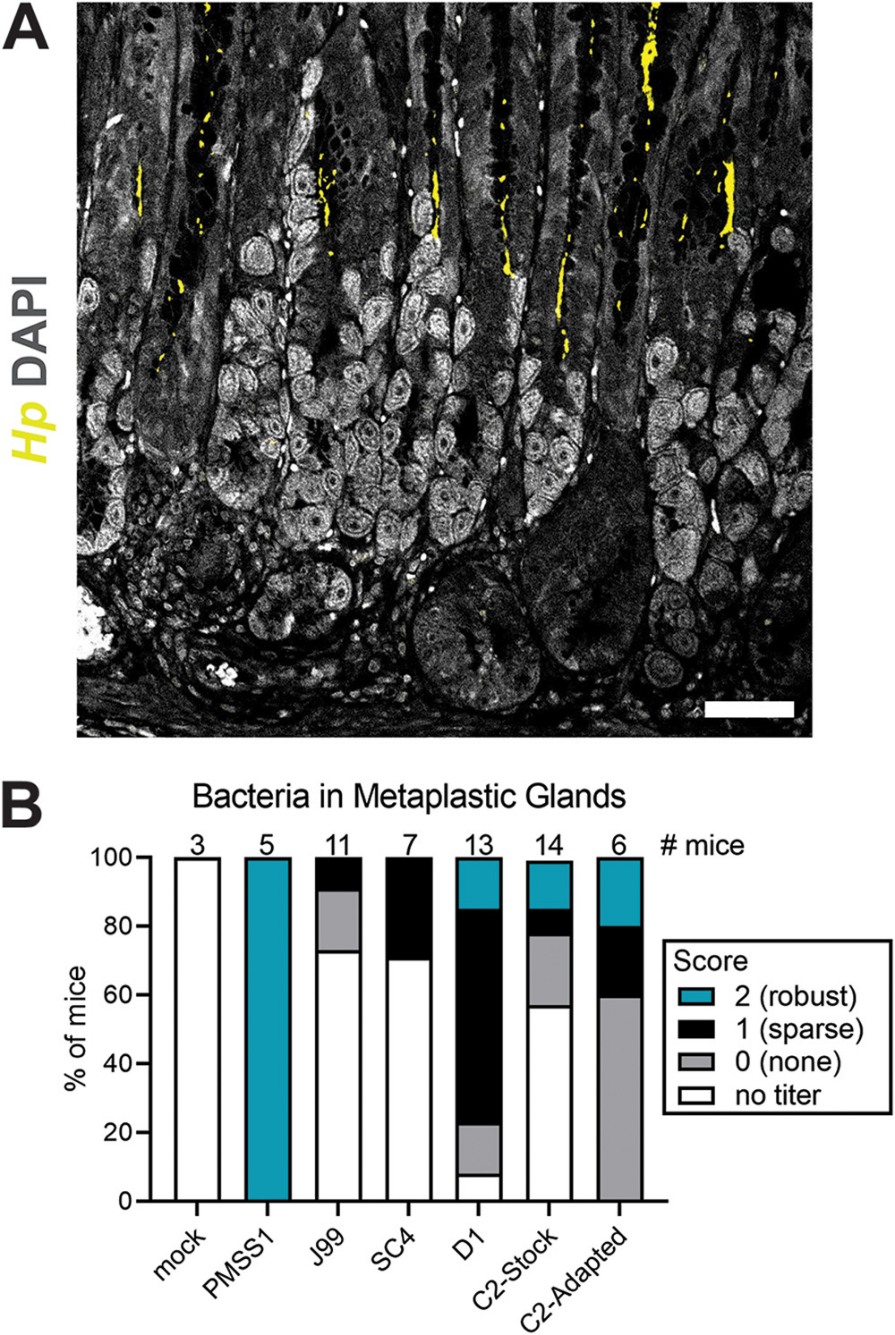
H. pylori strains can be detected in corpus glands at different densities. Thin stomach sections (4 μm) were stained for H. pylori and the corpus was assessed for bacterial colonization. (A) Shown is a representative image of a mouse infected with PMSS1 with a score of 2 (robust gland colonization). Yellow, *Hp*; gray, DAPI; scale bars, 50 μm. (B) Shown is the corpus gland colonization score for each H. pylori strain tested. The number of mice is given at the top of the graph. Blue indicates robust gland colonization, black indicates sparse gland colonization, and gray indicates no observable gland colonization. White indicates mice with undetectable *Hp* loads by gastric culture, which were not included in staining experiments.

Because PMSS1 exhibited robust colonization of corpus glands in KRAS+ mice (Fig. 1F), we tested whether other H. pylori isolates could also colonize KRAS+ corpus glands. We stained thin sections (4 μm) with an anti-H. pylori antibody (Fig. 3A) and semiquantitatively scored the extent of bacteria in glands in a blinded fashion, where 0 indicates no bacteria detected in corpus glands, 1 indicates few bacteria detected in the corpus (0 to 5 per field of view), and 2 indicates moderate to abundant bacteria detected in the corpus (>5 per field of view). Only mice with detectable CFU were included in these experiments. As expected, based on our analysis of thick sections (Fig. 1E and F), PMSS1 robustly colonized KRAS+ corpus glands (Fig. 3B and S1). Few bacteria of any strain tested were seen in the antral glands (not shown).

Most KRAS+ mice infected with J99 and SC4 had undetectable bacterial loads (Fig. 2A). In the few mice with detectable titers, few or no bacteria were observed within the glands (Fig. 3B). Thus, J99 and SC4 are poor colonizers of mice and poor colonizers of metaplastic glands. Interestingly, the D1 strain fared much better: only one mouse of 13 had an undetectable bacterial titer, and bacteria were observed in KRAS+ corpus glands in 10/13 mice (Fig. S2), while 2/13 mice had titers but did not have detectable bacteria in corpus glands. Once again, the C2-Stock strain gave rise to the most variable phenotypes. Eight of 14 mice had no titers and were excluded from the analysis, but of the remaining six, three had no bacteria observable in glands, one had sparse bacteria and two had abundant bacteria. Interestingly, this pattern was similar for the C2-Adapted strain. All eight mice had detectable titers (Fig. 2B). We randomly chose five mice for gland analysis and observed that three had no bacteria in glands whereas one had sparse bacteria and one had abundant bacteria. Thus, the C2-Adapted strain infects mice better than the C2-Stock strain does, but in mice with detectable titers, both strains colonize metaplastic glands to a similar extent.

10.1128/mbio.03116-22.3 FIG S2 Different H. pylori isolates have different propensities for colonizing KRAS+ glands. Representative images of corpus tissue are shown. Grey indicates nuclei and yellow indicates H. pylori cells. Arrowheads show examples of bacteria within glands. Scale bar, 100 μm. Download FIG S2, TIF file, 9.8 MB. Copyright © 2023 O’Brien et al. 2023 O’Brien et al. https://creativecommons.org/licenses/by/4.0/ This content is distributed under the terms of the Creative Commons Attribution 4.0 International license.

### H. pylori strains differentially impact metaplasia development.

**FIG 4:**
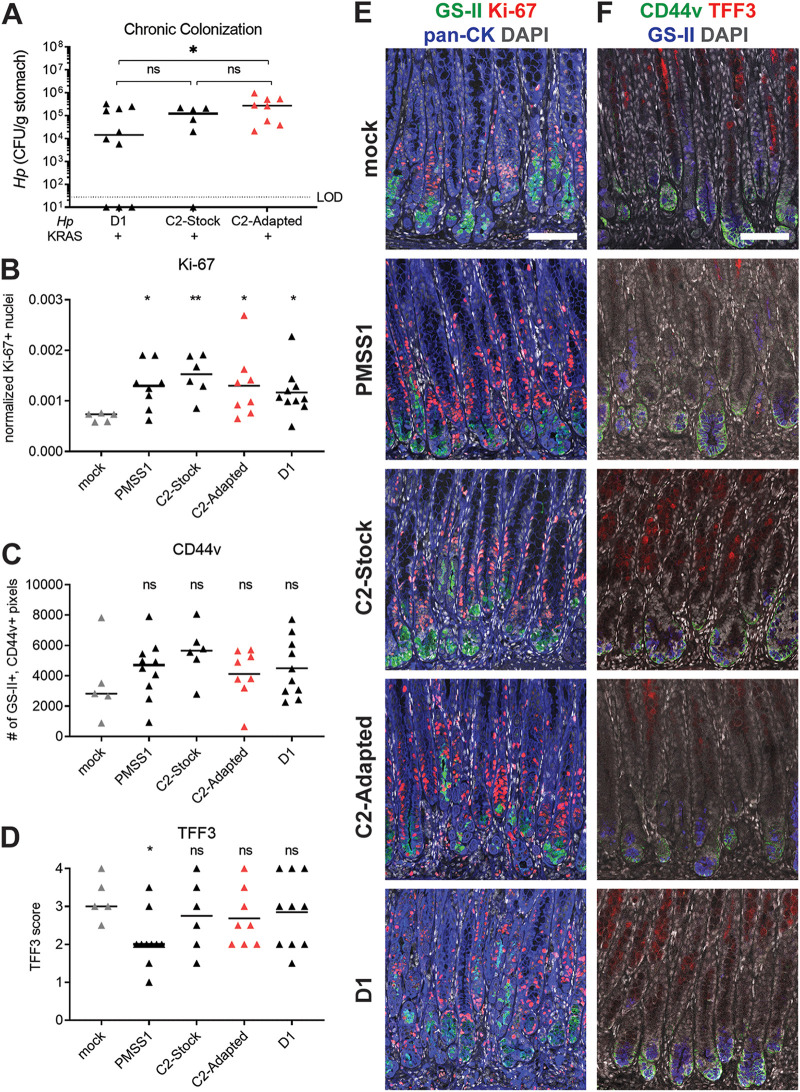
H. pylori isolates modulate expression of the metaplasia marker TFF3 in a strain-dependent manner. Mice were infected with the indicated H. pylori strains or mock-infected and active KRAS was induced. At 6 weeks, mice were euthanized and stomachs were assessed. (A) Stomach loads are shown. Data points represent actual values from each individual mouse, bars represent the median, and mice with no detectable CFU are plotted below the limit of detection (LOD). (B–F) IHC was used to detect expression of the indicated disease markers in thin stomach sections. Data are from N = 2–3 independent experiments. (B–D) Gastric metaplasia marker expression was assessed in mock-infected and H. pylori-infected mice. Three to five representative images per mouse were quantitatively or semiquantitatively assessed and the median value for each mouse is plotted. Bars on the graphs indicate the median value for each treatment group. *, *P < *0.05; **, *P* < 0.01, ns, not significant, Mann-Whitney U test. In B–D, each H. pylori group was compared against the mock-infected group. (B) Ki-67+/DAPI+ nuclei were enumerated and normalized to the DAPI content (total number of DAPI+ pixels) of each image. (C) The number of GS-II+, CD44v+ (dual-positive) pixels per image was quantified. (D) TFF3 staining was semiquantitatively scored in a blinded fashion. (E–F) Representative images are shown. Scale bars, 100 μm. (E) Stomachs were stained with antibodies against *K_i_*-67 (red) and pan-cytokeratin (blue), the lectin GS-II (green), and DAPI (gray). (F) Stomachs were stained with antibodies against CD44v (green) and TFF3 (red), the lectin GS-II (blue) and DAPI (gray).

We previously used *Mist1-Kras* mice to determine whether chronic infection with H. pylori strain PMSS1 impacted metaplasia development in KRAS+ mice, compared to mock-infected mice (14). In these experiments, mice are infected or mock-infected prior to tamoxifen administration to induce active KRAS. We found that at 6 weeks, the expression of SPEM markers and the cell proliferation marker Ki-67 differed between PMSS1-infected and mock-infected mice. Mice with concomitant PMSS1 infection and active KRAS had greater expression of Ki-67 and the SPEM marker CD44v10 (orthologous to human CD44v9, referred to herein as CD44v) and less expression of the IM marker TFF3 (14). Here, we tested whether strains that had evolved during colonization of the human stomach, D1 and C2, could elicit similar phenotypes. Mice were infected with D1, C2-Stock or C2-Adapted prior to induction of active KRAS and euthanized at 6 weeks (Fig. S3). The median stomach loads were similar among the different H. pylori strains (Fig. 4A) and similar to what we previously reported for PMSS1 (14). Interestingly, titers of the C2-Adapted strain were significantly greater than D1 titers. We noted that loads were lower after 6 weeks of infection than after 1 week (compare to Fig. 1B, 2A), consistent with the onset of adaptive immunity to infection.

10.1128/mbio.03116-22.4 FIG S3 Mouse model for assessing whether H. pylori strains impact metaplasia development. Mice are initially infected with various H. pylori strains or mock-infected. All mice receive tamoxifen (TMX) to induce active KRAS expression. After six weeks, mice are humanely euthanized. Stomachs are harvested and assessed by immunofluorescence microscopy analysis of disease marker expression, as well as H. pylori culturing and sequencing. Illustration created with BioRender.com. Download FIG S3, TIF file, 0.4 MB. Copyright © 2023 O’Brien et al. 2023 O’Brien et al. https://creativecommons.org/licenses/by/4.0/ This content is distributed under the terms of the Creative Commons Attribution 4.0 International license.

We next used immunohistochemistry (IHC) to assess the expression of Ki-67 (normalized to total DAPI content), CD44v and TFF3 in mouse corpus tissue. For comparison, we also tested KRAS+ mice with PMSS1 or mock infection (from a previous study (14) and two additional replicates for this study). In the corpus of healthy mice, Ki-67 is expressed at the isthmus of the glands (near the lumen); the lectin GS-II binds the mucin MUC6, which is expressed by cells in the neck region; and CD44v and TFF3 are not expressed. During metaplasia driven by active KRAS induction, Ki-67+ cells expand down from the isthmus; GS-II and CD44v label cells at the base of the glands; and TFF3 is expressed throughout the corpus. As we previously reported, Ki-67 and CD44v expression were elevated in mice with PMSS1 infection compared to mock-infected mice, whereas TFF3 expression was decreased (Fig. 4B to F). The other H. pylori strains also caused elevated Ki-67 and CD44v expression, demonstrating that these phenotypes may be a common response to H. pylori infection in the context of metaplasia. However, only H. pylori strain PMSS1 reduced TFF3 expression. Thus, some features of gastric preneoplastic progression are shared among different H. pylori strains, whereas other features may be strain-dependent.

### Sequence comparison of D1 and C2 strains reveals mutations in outer membrane proteins.

Isolates from the J99 collection exhibited differential colonization patterns in KRAS+ and KRAS− mice (Fig. 2A), despite these strains having relatively few polymorphisms compared to isolates from different individuals; that is to say, D1 is more genetically similar to C2 than it is to PMSS1, for example. In order to investigate potential genetic determinants of colonization, we used our previously reported whole-genome sequencing data (27) to examine the 349 polymorphic loci between isolates D1 and C2. For this study, we focused on the 175 polymorphisms that resulted in alterations to amino acid sequence, which includes 123 nonsynonymous single nucleotide polymorphisms (SNPs) and 52 indels detected across 68 genes, including outer membrane proteins (OMPs) and predicted OMPs.

The H. pylori genome is enriched for repetitive regions, particularly in the genes encoding OMPs, which allow for high rates of recombination (29, 30). Therefore, we predicted that many of these alterations reflect inter- or intragenomic recombination events or mis-mapping of short reads to paralogs with high sequence identity. We leveraged ClonalFrameML to bioinformatically predict genes with mutations predicted to be introduced through importation of divergent DNA (31). As expected, more than half of these alterations (65%) were predicted to have been introduced via recombination events and were detected across only 14 genes. The remaining 46 SNPs and 16 indels differentiating C2 and D1, which are not predicted to arise from recombination, are listed in Table S1 and S2.

10.1128/mbio.03116-22.6 TABLE S1 Nonsynonymous SNPs (n = 46) between isolates D1 and C2 stock. Download Table S1, PDF file, 0.01 MB. Copyright © 2023 O’Brien et al. 2023 O’Brien et al. https://creativecommons.org/licenses/by/4.0/ This content is distributed under the terms of the Creative Commons Attribution 4.0 International license.

10.1128/mbio.03116-22.7 TABLE S2 Insertion or deletion events (n = 16) between isolates D1 and C2 stock. Download Table S2, PDF file, 0.01 MB. Copyright © 2023 O’Brien et al. 2023 O’Brien et al. https://creativecommons.org/licenses/by/4.0/ This content is distributed under the terms of the Creative Commons Attribution 4.0 International license.

To further address the limitations of mapping short read data to extended repeats in the reference, we generated long read assemblies of isolate D1 using Pacific Biosciences (PacBio) single-molecule real-time (SMRT) sequencing technology (32). Long read assemblies revealed that some of these polymorphisms predicted to be introduced through recombination were a result of short reads misaligning to the reference strain in repetitive regions. However, 10 of the 14 genes did have mutations or significant structural differences that were detected using both short and long read data (Table S3). Six out of 10 encode outer membrane proteins: *sabA*, *sabB*, *homA*, *hopJ*, *hopK, and hopQ*. Genes jhp0440, jhp1031, and *hopQ* had significantly altered protein lengths. Isolate D1 had an alternate start site rendering the protein 17 amino acids shorter than the C2 HopQ protein.

10.1128/mbio.03116-22.8 TABLE S3 Variation between C2 and D1 predicted to be the result of recombination. Download Table S3, PDF file, 0.01 MB. Copyright © 2023 O’Brien et al. 2023 O’Brien et al. https://creativecommons.org/licenses/by/4.0/ This content is distributed under the terms of the Creative Commons Attribution 4.0 International license.

### The *sabB* locus undergoes dynamic gene conversion events *in vivo*.

**FIG 5:**
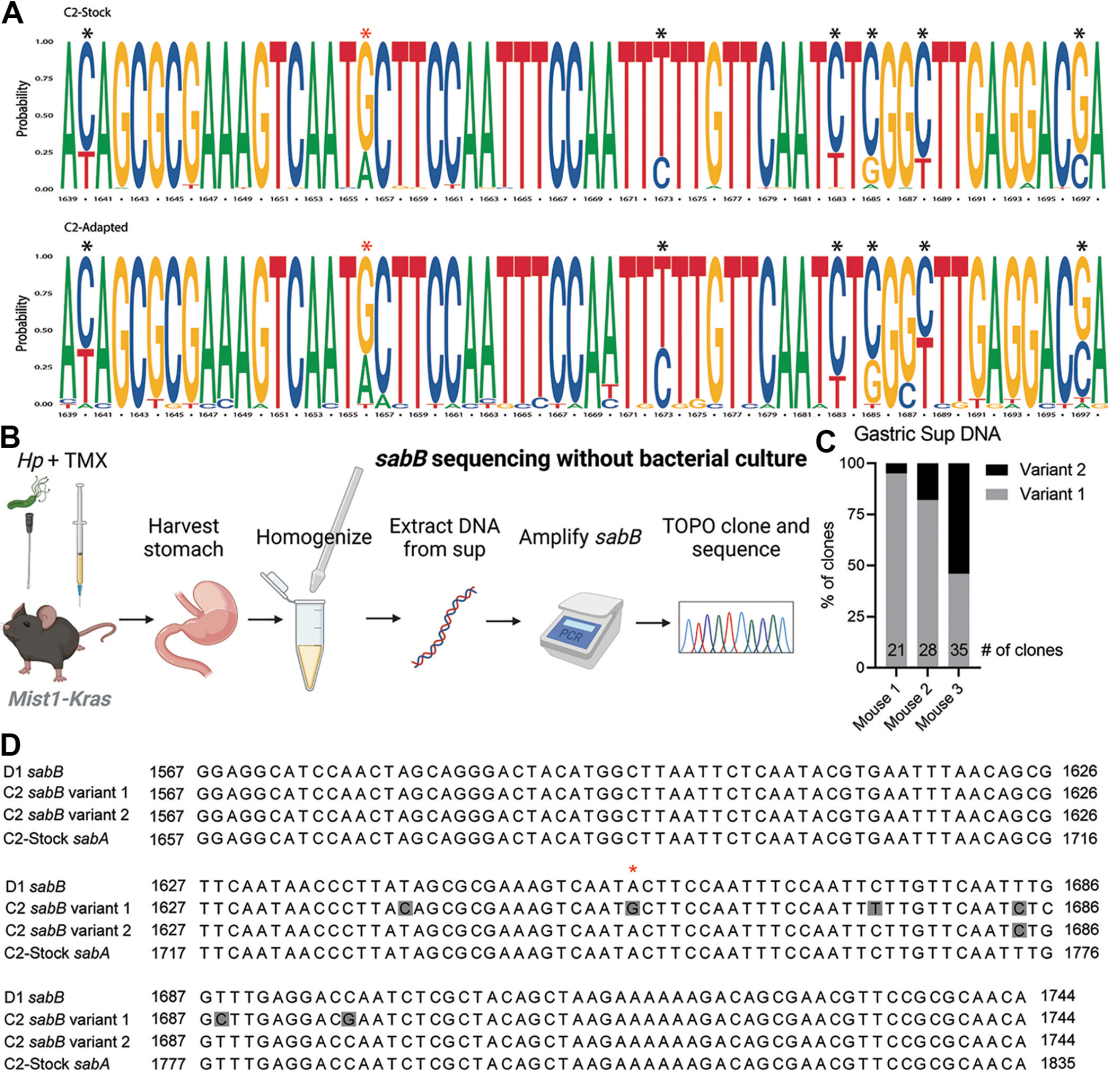
The C2 strain is a mixed population with two alleles at the *sabB locus* that change in frequency during *in vivo* infection. (A) Sequence logos depict the probability of each base appearing at each position of the C-terminal region of *sabB* in C2-Stock (top) and C2-Adapted (bottom). Asterisks highlight positions with nucleotide variants. The red asterisk indicates the nonsynonymous change. (B) Schema for amplifying and sequencing *sabB* from mouse gastric homogenate supernatant. Illustration created with BioRender.com. (C) The proportion of TOPO clones of *sabB* variant 1 (gray) and variant 2 (black) is depicted for gastric homogenate supernatants from the indicated mice infected with C2-Stock for 6 weeks. (D) The sequence alignment shows the C-terminal region of *sabB* in strains D1, C2 variant 1, and C2 variant 2, with nucleotide variants highlighted in gray. The red asterisk indicates a nonsynonymous change. The homologous region of *sabA* from C2-Stock is also shown.

C2-Adapted, which colonized the metaplastic stomach more robustly than C2-Stock (Fig. 2B), was also sequenced using the PacBio SMRT sequencing platform to test if genetic changes occurred during acute colonization of the metaplastic stomach environment. Notably, the only region that differed between the two strains was the C terminus of the outer membrane protein SabB. The consensus sequence of C2-Adapted differed from C2-Stock at five positions, all within a short 50 nucleotide region. Only one of these polymorphisms results in an amino acid change: a mutation from threonine, an amino acid with a polar side chain, to alanine, an amino acid with a hydrophobic side chain, at position 553 (Fig. 5A, red asterisk). Further analysis of the individual C2-Stock Illumina reads revealed that the five polymorphisms detected in C2-Adapted strain were present at a low frequency, indicating that there may be two alleles present in this population (Fig. 5A). The predominant allele (variant 1) is estimated to be present at a frequency of ~80% compared to 20% frequency of the minor allele (variant 2). Similar analysis of the PacBio sequencing reads demonstrated that in the C2-Adapted strain, variant 2 increased in abundance to ~40 to 50% (Fig. 5A). Therefore, we hypothesized that variant 2 was selected for during gastric infection.

Attempts to use Sanger sequencing to detect the *sabB* variant 2 in genomic DNA isolated from either the C2-Stock or Adapted strains were unsuccessful, possibly because culturing the bacteria on plates prior to DNA extraction selected for variant 1. To circumvent this issue, we isolated DNA directly from gastric homogenate supernatants (Fig. 5B) from mice infected with the C2-Stock strain for 6 weeks (from Fig. 4A). After PCR amplification of *sabB*, we used TOPO cloning and sequencing to test for the frequencies of variant 1 and 2 in each mouse. We found that the frequency of variant 2 ranged from 5 to ~50% in different mice, confirming there are two alleles of *sabB* present in the C2-Stock and that this genetic variation is dynamic during *in vivo* colonization (Fig. 5C).

SabB has paralogs SabA and HopQ, which facilitate binding to sialic acid and CEACAMs, respectively (33, 34). Gene conversion events among these adhesins and putative adhesins are thought to modulate adherence to inflamed tissue during chronic infection (35, 36). All five SNPs in C2 *sabB* variant 2 were shared with the homologous region of *sabA*, suggesting that a recombination event gave rise to *sabB* variant 2 (Fig. 5D). Strikingly, the *sabB* C-terminal region from the D1 strain also shared the five SNPs with C2 variant 2 and *sabA*, though D1 SabB was overall highly divergent from C2 SabB (Fig. S4). Thus, variant 2 of *sabB* is associated with robust mouse infection and likely arose from a *sabA* recombination.

10.1128/mbio.03116-22.5 FIG S4 The outer membrane protein SabB differs between PMSS1 and strains from the J99 collection. Shown is a sequence alignment of the SabB protein comparing PMSS1 with D1, J99, and C2 variants 1 and 2. Amino acid changes are indicated. The C-terminal region of interest from Fig. 5D is indicated by the red line. Download FIG S4, TIF file, 11.8 MB. Copyright © 2023 O’Brien et al. 2023 O’Brien et al. https://creativecommons.org/licenses/by/4.0/ This content is distributed under the terms of the Creative Commons Attribution 4.0 International license.

### *SabB* promotes tissue adherence and colonization of the mouse stomach.

**FIG 6:**
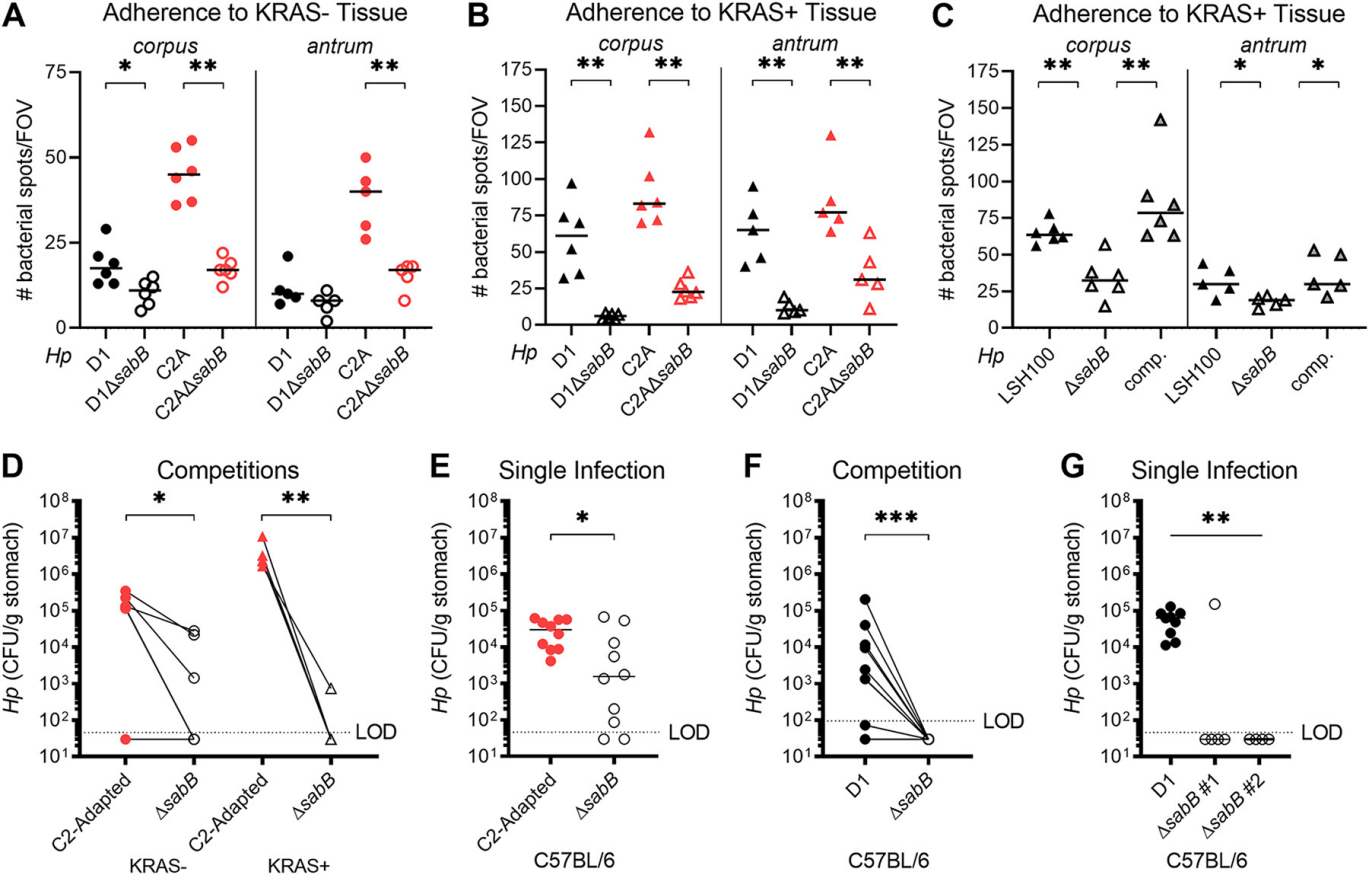
SabB promotes colonization of healthy and metaplastic stomach by the D1 and C2-Adapted strains. (A–C) Tissue adherence was assessed using a previously described *ex vivo* binding assay (36). The indicated H. pylori strains were labeled with fluorescein isothiocyanate (FITC), applied to mouse tissue sections and the number of bacterial spots per field of view (FOV) was quantified. Each data point indicates one FOV and bars indicate the median value. Two to three technical replicates were performed with bacteria labeled on two different days and a representative experiment is shown. (A) The indicated strains were adhered to KRAS− tissue. C2A, the C2-Adapted strain. (B–C) The indicated strains were adhered to KRAS+ tissue. (C) A *sabB* mutation (Δ*sabB*) was generated in a derivative of G27 (LSH100) and complemented (“comp.”) at a neutral intergenic locus. (D–F) Single infections and competitions were performed to compare wild-type and isogenic Δ*sabB* strains. (D) In *Mist1-Kras* mice, active KRAS was induced or sham-induced. 8 weeks later, mice were infected with a 1:1 mixture of C2-Adapted and an isogenic Δ*sabB* mutant for 1 week. (E–G) C57BL/6 mice were infected with the indicated strains for 1 week. Data are from N = 2 independent experiments. Data points represent actual values from each individual mouse, bars represent the median, and mice with no detectable CFU are plotted below the limit of detection. In D and F, lines connect wild-type and mutant titers from the same mouse. *, *P < *0.05; **, *P < *0.01; ***, *P < *0.001. For A–F, statistical significance was assessed with a Mann-Whitney U test; for G, significance was assessed with a Kruskal-Wallis test.

Because our *in silico* analysis suggested that *sabB* may be important during infection, we investigated this gene further. To test whether SabB, like its paralogues, could promote adherence of H. pylori to gastric tissue, we generated Δ*sabB* mutants in the C2-Adapted and D1 strains and tested their ability to adhere to *ex vivo* gastric tissue compared to the parental strains. In KRAS− gastric tissue, adherence of both parental strains was greater than adherence of the isogenic Δ*sabB* mutants, both in the corpus and the antrum (Fig. 6A). The same was true in KRAS+ stomachs (Fig. 6B), and notably, the parental strains bound about twice as well to KRAS+ tissue than to KRAS− tissue. Interestingly, the C2-Adapted strain was more adherent than D1 in KRAS− tissue, but the two strains were similar in KRAS+ tissue. We also generated a Δ*sabB* mutation in a derivative of G27, a highly tractable model H. pylori strain (37). Loss of *sabB* in this strain background also significantly reduced adherence to KRAS+ corpus and antrum tissue, and the defect could be rescued by expressing *sabB* at a neutral intergenic locus (38) (Fig. 6C).

Finally, to test whether SabB is necessary *in vivo*, we infected mice with the Δ*sabB* mutants and/or parental strains. First, 8 weeks after active KRAS induction or sham induction, mice were infected with a 1:1 mixture of the C2-Adapted strain and Δ*sabB* mutant (Fig. 6D). The Δ*sabB* mutant was somewhat attenuated in KRAS− mice and highly attenuated in KRAS+ mice, demonstrating that SabB promotes gastric colonization. Because the Δ*sabB* mutant had a phenotype in KRAS− (i.e., healthy) mice, we used C57BL/6 mice to further probe the role of SabB in gastric infection. The C2-Adapted Δ*sabB* mutant performed worse than the parental strain in single infection (Fig. 6E), demonstrating that its *in vivo* attenuation occurs whether or not the wild-type strain is present. Strikingly, the D1Δ*sabB* strain was not recovered from any mice in competitive infections (Fig. 6F) and only from one out of five mice in single infections (Fig. 6G). A second clone was not recovered from any mice (Fig. 6G), which suggests that the colonization defect was due to the lack of *sabB*, not due to an off-target effect with the constructed strain. Taken together, these findings demonstrate that SabB promotes H. pylori adherence to tissue and stomach infection.

## DISCUSSION

It has been well established that prolonged H. pylori infection can induce premalignant tissue changes, but little is known about the dynamics between host and pathogen at later stages of disease development. In this study, we used the *Mist1-Kras* mouse model to examine H. pylori strain-specific determinants of stomach and gland colonization during the development of gastric metaplasia. We found that H. pylori strains have different propensities for infecting the stomach with and without metaplasia (+/− KRAS). Strains that can robustly colonize KRAS+ mice have an expanded niche in the corpus glands, similar to what is observed with long term (>4 wks) chronic infections in mice (8, 10). The putative adhesin SabB promotes colonization of both the healthy and metaplastic stomach environment and seems especially important in the metaplastic (KRAS+) tissue environment. Furthermore, the ability of H. pylori to infect KRAS+ mice can be selected for during acute infection of metaplastic tissue via changing *sabB* allele frequencies.

J99 strains, originating from the same patient at two different stages of disease (25, 26), exhibited differential colonization in KRAS+ mice. J99 is a relatively poor colonizer of both KRAS− and KRAS+ mice. SC4, isolated at the early time point but belonging to a different genetic subgroup from J99, was also a relatively poor colonizer of KRAS +/− mice. D1, collected from the patient during a period of gastric atrophy, robustly colonized both KRAS− and KRAS+ mice, and titers were greater in KRAS+ stomachs than KRAS− stomachs. In contrast, C2, originating from the same time point as D1, had relatively poor colonization in some mice but very robust colonization in others. We showed that C2 could be adapted to *in vivo* colonization through just 1 week of infection in the KRAS+ stomach by selecting for a lower frequency allele at the *sabB* locus.

Not all of the clinical isolates we tested colonized the metaplastic stomach of KRAS+ mice, indicating there are bacterial determinants of metaplastic colonization. The host environment where clinical strains were isolated from may make certain isolates better adapted for colonization. Complete genome sequencing suggests that changes in the outer membrane protein SabB facilitated quick adaptation to the metaplastic stomach environment in isolate C2.

Prior to this study, little was known about the role of SabB. Work by other groups has suggested that *sabB* presence and phase variation state contribute to increased host expression of sirt7 (39) and PREX2 (40), as well as increased mouse colonization (41). It has also been suggested that “off” status of *sabB* through phase variation is associated with increased likelihood of duodenal ulcers (42). However, our study suggests that *in vitro* growth can alter the genotype of *sabB*, which may influence the data underlying some of these conclusions.

SabB is a protein that is part of a paralogous family, including HopQ, which binds to CEACAMs, and SabA, which binds to sialic acid (33, 34). SabA was previously reported to mediate H. pylori interactions with SPEM (metaplastic) cells (43), suggesting that this protein family is an important tool in H. pylori’s toolbox for late disease-state persistence. These proteins are some of the most highly variable in the H. pylori genome and can recombine to quickly adapt to changes to the gastric environment (36, 44). Gene conversion events and allelic diversity in *sabB* have been identified in several whole-genome sequencing data sets of human H. pylori isolates (16, 17, 36). The altered bases within *sabB* variant 2 align to the paralog *sabA* from the C2-Stock strain, indicating that these changes were most likely introduced through an intragenomic recombination event between *sabA* and *sabB*. Inflamed tissue is more likely to express sialylated glycoconjugates than healthy tissue is (34), which may suggest that H. pylori genetically alters *sabA* and *sabB* in order to modulate binding to inflamed tissue. Our data suggest that *sabB* may play a previously unknown role in achieving colonization of tissue that is inflamed or exhibiting SPEM. However, it is likely not the only player. Other differences between C2 and D1 include mutations in two fucosyltransferases, *fucA* and *fucU*, which are involved in modifying bacterial lipopolysaccharide to mimic surface sugars on the host epithelium in order to evade immune detection (45); and several mutations in genes involved in DNA uptake, metabolism, and repair, including *comB8*, *comM*, *hsdR*, *hsdM*, and *hsdS*.

In humans, H. pylori triggers chronic gastritis. In a subset of individuals, this gastric inflammation leads to gastric atrophy (loss of acid-producing parietal cells), which can then progress to metaplasia, dysplasia, and finally cancer. Once this pathogenic cascade is initiated, it is thought that H. pylori does not further contribute to the development of cancer and that these tissue alterations are not favorable to H. pylori proliferation. This is referred to as the “hit and run” theory of gastric cancer development (46). This implies eradication of H. pylori infection is unnecessary once metaplastic lesions have developed. However, a long-term study with a Colombian cohort shows that eradication of H. pylori after discovery of metaplastic legions reduces risk of gastric cancer, suggesting a role for H. pylori in the development or acceleration of gastric cancer (47). Host-pathogen interactions during premalignant tissue alterations are understudied, but we recently showed that H. pylori interacts with tissue in ways that impact disease progression and skew the immune response (14). In another mouse model of gastric atrophy and SPEM, induced via chemical ablation of parietal cells, H. pylori was shown to preferentially interact with SPEM cells within the corpus glands (43). Notably, KRAS+ mice have moderate gastric inflammation marked by M2 macrophage expansion (14, 19). Here, we show that certain strains of H. pylori readily colonize the metaplastic stomach despite this preexisting inflammatory milieu, which might have been predicted to reduce or even clear infection. Furthermore, some H. pylori strains exhibited an expanded niche in the corpus glands. Together these data suggest that metaplastic environments may be suitable or even preferable for H. pylori growth and challenge the hit-and-run theory of carcinogenesis. This new model provides avenues to further explore H. pylori interactions with metaplastic tissue and cell types specific to metaplastic glands.

A limitation of our study is that our imaging methods, whether using thick or thin sections of tissue, do not capture the bacteria in the mucus layer above the glands, because the surface mucus does not withstand fixation and sectioning. Culturing of stomach homogenates captures bacteria in both the glands and the mucus, and when we cultured the corpus and antrum separately, we only recovered bacteria from the corpus. The relatively high limit of detect in that experiment may have precluded us from culturing bacteria from the antrum. However, in both thick and thin sections of KRAS+ mice, we predominantly observed H. pylori cells in the corpus. More work is needed to characterize the role of antrum colonization in this model and whether SabB contributes.

It is remains unknown if H. pylori has evolved to exploit preneoplastic tissue changes such as SPEM, IM, and dysplasia in order to expand its niche and increase transmission, or if preneoplasia is an unintended consequence of bacterial growth that is tolerated by, but not advantageous to, H. pylori. However, our findings suggest that certain bacterial strain-specific factors may promote colonization of the metaplastic stomach and these factors can vary among isolates in a single infected individual during the course of disease development. Additional studies of bacterial genetic factors facilitating colonization during SPEM may identify important predictors for risk of transmission and/or disease progression.

## MATERIALS AND METHODS

### Ethics statement.

All mouse experiments were performed in accordance with the recommendations in the National Institutes of Health Guide for the Care and Use of Laboratory Animals. The Fred Hutchinson Cancer Center is fully accredited by the Association for Assessment and Accreditation of Laboratory Animal Care and complies with the United States Department of Agriculture, Public Health Service, Washington State, and local area animal welfare regulations. Experiments were approved by the Fred Hutch Institutional Animal Care and Use Committee, protocol number 1531.

### H. pylori culture.

The H. pylori strains used in this study were PMSS1 (20), 7.13 (48), the G27 (49) derivatives NSH57 (23) and LSH100 (37), and four representative isolates from the J99 culture collection (25, 27, 29): J99, SC4, D1 and C2. All H. pylori isolates were grown on solid media, horse blood agar (HB agar). HB agar plates contain 4% Columbia agar base (Oxoid), 5% defibrinated horse blood (Hemostat Labs), 10 mg/mL vancomycin (Thermo Fisher), 2.5 U/mL polymyxin B (Sigma-Aldrich), 8 mg/mL amphotericin B (Sigma-Aldrich), and 0.2% β-cylodextrin (Thermo Fisher). For HB agar plates used to grow H. pylori from homogenized mouse stomach, 5 mg/l cefsulodin (Thermo Fisher), 5 mg/l trimethoprim (Sigma) and 0.2 mg/mL of bacitracin (Acros Organics, Fisher) were added to prevent outgrowth of mouse microflora. For competition experiments, 15 μg/mL chloramphenicol was added to distinguish between mutant (chloramphenicol-resistant) and wild-type (chloramphenicol-sensitive) bacteria. Shaking liquid cultures were grown in BB10, Brucella broth (Thermo Fisher) supplemented with 10% heat-inactivated FBS (Gemini BioProducts). H. pylori on plates and in liquid culture were grown at 37°C in a microaerobic conditions (10% CO_2_, 10% O_2_, and 80% N_2_) using a tri-gas incubator.

### *Mist1-Kras* mouse model.

All experiments used Mist1-CreERT2 Tg/+; LSL-K-RAS G12D Tg/+ (“*Mist1-Kras*”) mice described previously (19) or C57BL/6J mice as indicated. Mice were housed in sterilized microisolator cages with irradiated rodent chow, autoclaved corn cob bedding, and acidified, reverse-osmosis purified water. Mice were genotyped from ear punches as previously described (19). Expression of the KRAS transgene was induced via subcutaneous administration of 5 mg of tamoxifen (Sigma-Aldrich) in corn oil (Sigma-Aldrich) for three consecutive days. Sham induced mice were administered corn oil subcutaneously on the same schedule. All mouse experiments were performed as previously described (14). The inoculum for each infection was 5×10^7^ cells of the indicated strain or strains. After stomach excision, the forestomach was removed, and the stomach was opened along the lesser curvature. Stomachs were divided into equal thirds or halves containing both antral and corpus regions. For culture, stomach portions were placed in 0.5 mL of sterile BB10 media, weighed, and homogenized. Serial homogenate dilutions were plated on nonselective HB plates, or both nonselective and chloramphenicol-containing plates for competition experiments. Homogenates were then pelleted at 15,000 rpm in a benchtop centrifuge (Eppendorf 5424) and the supernatant was stored at −20°C. After 5 to 9 days in tri-gas incubator, CFU (CFU) were enumerated and reported as CFU per gram of stomach tissue.

### Gland occupation analysis.

PMSS1 gland colonization was assessed as previously described (8) with the following modifications. One third of the stomach was embedded in 4% agarose in 1× phosphate-buffered saline (PBS, Gibco) and cut into 200 μm thick longitudinal sections using a Leica VT1200S Vibratome. Tissue sections were then permeabilized overnight at 4°C by gently rocking in blocking buffer comprising 3% bovine serum albumin (Sigma-Aldrich), 1% saponin (Sigma-Aldrich) and 1% Triton X-100 (Sigma-Aldrich) in phosphate-buffered saline (PBS). Stomachs were incubated with 1:1,000 rabbit polyclonal anti-H. pylori PMSS1 antibody (gift of Manuel Amieva, Stanford University) and 1:2,000 GS-II 488 (conjugated lectin from Griffonia simplicifolia, Fisher) for 2 h at 4°C with gentle rocking. After three 10-minute washes in PBS, samples were incubated with 1:2,000 Alexa Fluor 647 donkey anti-rabbit IgG (Invitrogen) and 1:2,000 DAPI for 2 h at room temperature with gentle rocking. After three 10-minute washes in PBS, sections were mounted onto glass slides with imaging spacers cut from Parafilm (Bemis) in ProLong Diamond Anti-Fade Reagent (Molecular Probes) and coverslips were sealed with VaLP (1:1:1 Vaseline:Lanolin:Paraffin). Stomach sections were imaged on a Zeiss LSM 780 laser-scanning confocal microscope, or with an UltraView spinning disk microscope (PerkinElmer). Z-stacks were collected to visualize H. pylori within glands and assessed in Volocity (Quorum Technologies) to enumerate H. pylori in glands based on fluorescent voxels as previously described (8).

### Immunohistochemistry to assess tissue markers.

Immunohistochemistry was performed as previously described (14). Briefly, thin sections of formalin-fixed, paraffin-embedded tissue were deparaffinized with Histo-Clear solution (National Diagnostics) and rehydrated in decreasing concentrations of ethanol. Slides were boiled in a pressure cooker for 15 min in Target Retrieval Solution (Agilent Dako) for antigen retrieval. Slides were incubated with Protein Block, Serum Free (Agilent Dako) for 90 min at room temperature. Primary antibodies (Table S4) were diluted in Protein Block, Serum Free, or Antibody Diluent, Background Reducing (Agilent Dako), and applied to the slides overnight at 4°C. Secondary antibodies were diluted 1:500 in Protein Block, Serum Free and slides were incubated for 1 h at room temperature protected from light. Slides were mounted in ProLong Diamond antifade reagent with DAPI (Invitrogen) and allowed to cure for 24 h at room temperature before imaging. Slides were imaged on a Zeiss LSM 780 laser-scanning confocal microscope using Zen software (Zeiss). For assessment of H. pylori gland colonization, the entire length of each corpus was inspected for H. pylori cells and three to five representative images per sample were taken. For assessment of epithelial disease markers (Ki-67, CD44v, TFF3), three to five representative images of the corpus were taken per sample and samples were quantified as previously described (14).

10.1128/mbio.03116-22.9 TABLE S4 Antibodies and lectins used to assess *H. pylori* gland colonization and expression of epithelial disease markers. Download Table S4, PDF file, 0.01 MB. Copyright © 2023 O’Brien et al. 2023 O’Brien et al. https://creativecommons.org/licenses/by/4.0/ This content is distributed under the terms of the Creative Commons Attribution 4.0 International license.

### PacBio long read sequencing.

Single molecule real-time sequencing (SMRT-Seq) was carried out on a PacBio Sequel-I instrument (Pacific Biosciences, USA). Genomic DNA to be sequenced was purified using the Wizard Genomic DNA purification kit (Promega), concentration was determined using the Qubit dsDNA HS (High Sensitivity) assay kit (Thermo Fisher), and purity was calculated using a NanoDrop One (Thermo Fisher). Genomic DNA samples (3 μg) were sheared to an average size of 12 kb via G-tube (Covaris) before library preparation. Libraries were then generated with SMRTbell Express Template Prep kit 2.0m and pooled libraries were size-selected using the BluePippin system (Sage Sciences) at a 4 kb minimum threshold. Sequencing reads for the D1 strain were processed using the Pacific Biosciences SMRTAnalysis pipeline version 8.0.0.80529 and assembled using Microbial Assembler. Genome assembly showed 21,312 polymerase reads that were further partitioned into 210,582 subreads with an N50 value of 5,441 nucleotides and a total number of subread bases of 767,735,588 with a mean coverage of 397×. Genome assembly of D1 resulted in a single contig: a chromosomal sequence of 1,685,094 bp. Sequencing reads for the C2-Adapted strain were processed using the Pacific Biosciences SMRTAnalysis pipeline version 8.0.0.80529 and assembled using Flye *de novo* assembler (50). Genome assembly showed 5,657 polymerase reads that were further partitioned into 41,337 subreads with an N50 value of 6,850 nucleotides and a total number of subread bases of 141,837,408 with a mean coverage of 69×. Genome assembly of the C2-Adapted strain resulted in a single contig: a chromosomal sequence of 1,653,204 bp.

### Bioinformatics.

Short reads for isolates C2-Stock and D1 were downloaded from the NCBI SRA database (BioProject accession: PRJNA622860). Single nucleotide variants differentiating D1 and C2-Stock were determined by aligning short reads to J99 reference (AE001439) with BreSeq v0.35.0 software using default parameters (51). ClonalFrameML was used to predict putative sites of recombination (31). The D1 and C2-Adapted isolates were sequenced on a PacBio Sequel instrument to generate long sequence reads. Closed reference sequences were generated using either SMRT Link web-based analysis suite (https://www.pacb.com/support/software-downloads/) or Flye (https://github.com/fenderglass/Flye) assembly pipelines and genomes were annotated using Pathosystems Resource Integration Center (PATRIC, [https://www.patricbrc.org]) annotation tool with Helicobacter pylori (species ID: 210) as the reference. Recombination events predicted by short read assemblies were validated by cross comparing with sequences generated from long read assemblies. Genomic comparisons of C2-Stock versus adapted isolates were performed by aligning C2-Stock short reads with Breseq v0.35.0. using C2-Adapted PacBio assembly as the reference. The frequency matrix of each base at each position for the short read sequencing was generated using pileup2acgt from the Sequenza package on the previously reported SAMtools pileup (27, 52, 53). The frequency matrix for the long read data was generated by mapping the reads to the Flye assembly using Minimap2 (54) and running the output through bam-readcount (https://github.com/genome/bam-readcount). The sequence logos were generated with ggseqlogo (https://github.com/omarwagih/ggseqlogo) from ggplot2 (55).

### TOPO cloning and sequencing.

DNA was isolated from stored mouse gastric homogenate supernatants via enzymatic digestion and phenol-chloroform extraction (56). The C-terminal region of *sabB* was amplified by PCR using primers 5’aagctcaaggcaatctctgtgc3’ (sabBFor) and 5’gatcatgcgtttttgatccctgg3’ (jhp0660R) (36). The PCR product was verified by agarose gel electrophoresis and used for TOPO cloning with the Zero Blunt TOPO PCR Cloning kit (Invitrogen). After selection on kanamycin, clones were screened by colony PCR with sabBFor and jhp0660R primers and Go *Taq* master mix (Promega). Dye-terminator sequencing using BigDye (Thermo Fisher) with the jhp0660R primer was performed by the Fred Hutch Genomics Shared Resource and the results were analyzed using SnapGene software version 5.2.4 (Insightful Science).

### Construction of H. pylori mutants.

Deletion mutants of *sabB* (Δ*sabB*) were constructed in four H. pylori strain backgrounds (D1, C2-Adapted, LSH100 *rdxA::aphA3sacB*, and LSH100 *hp0203-0204* intergenic region::*sabB*). Deletions of *sabB* from D1 and C2-Adapted were generated by transforming parent strains with gDNA from J99 *sabB::catsacB* (36). The gDNA was extracted using a Wizard Genomic DNA purification kit (Promega). Clones were selected on HB plates with 15 μg/mL chloramphenicol as previously described (57, 58). Clones were confirmed using diagnostic PCR and Sanger sequencing with primers 5′ tgggttgagatcatgcaagcat 3′ (jhp0658F) and 5′ gatcatgcgtttttgatccctgg 3′ (jhp0660R) (36). LSH100 *rdxA::aphA3sacB sabB::catsacB* was generated with the same strategy but using gDNA extracted from D1Δ*sabB*. Strains were back-crossed to reduce the likelihood of off-target mutations. To complement the mutation, *sabB* was amplified from strain D1 using primers McGee_sabB_fwd (5′ tagaactagtggatccattttcatttctattcatgtttacaataaaaaaattactttaag 3′) and McGee_sabB_rev (5′ atcgataagcgaattcttaataagcaaacacataattgagatacacgctataaagc 3′) and cloned into the pDYC40 plasmid that contains a kanamycin resistance cassette (59) via In-Fusion cloning (TaKaRa). This plasmid is designed for complementation at a previously characterized, neutral intergenic chromosomal site (*hp0203-0204* intergenic region) (38). LSH100 was transformed with the resulting pDYC40::*sabB* plasmid and clones were selected on HB agar with 25 μg/mL kanamycin. The native *sabB* locus was then mutated by transforming with a PCR product prepared from D1Δ*sabB* using primers jhp0658F and jhp0660R to amplify the *catsacB*-interrupted *sabB* region and selecting on HB agar with 15 μg/mL chloramphenicol. All mutants were confirmed with PCR and Sanger sequencing.

### *Ex vivo* tissue adherence assay.

The *ex vivo* tissue adherence assay was performed as previously described (36). Briefly, bacteria used for the assay were grown in 10 to 20 mL of BB10 (10% FBS in Brucella broth) overnight to achieve an optical density at 600 nm (OD_600_) between 0.5 and 1.0. Bacteria were pelleted by centrifuging and then washed in PBS-T (0.05% Tween 20 in phosphate-buffered saline). Bacteria were resuspended in carbonate buffer and incubated with fluorescein 5(6)-isothiocyanate (FITC, Sigma) dissolved in DMSO (dimethyl sulfoxide, Alfa Aesar) for 10 min and washed in oxidized 1% bovine serum albumin (BSA). The labeled organisms were stored in oxidized 1% BSA at −80°C. Slides with mouse stomach tissue sections were deparaffinized in Histo-Clear solution (National Diagnostics) and rehydrated with isopropanol, ethanol, deionized water, and PBS. Slides were blocked with oxidized 1% BSA for two and a half hours in a hydration chamber before incubating with FITC-labeled bacteria (OD_600_ = 0.01) for another 2 h. The excess bacteria were washed off in PBS-T and the slides were mounted in ProLong Diamond antifade reagent with DAPI (Invitrogen). The slides were allowed to cure for 24 h at room temperature before imaging at ×400 magnification on a Zeiss LSM 780 laser-scanning confocal microscope using Zen software (Zeiss). For the quantification of FITC-labeled bacteria adhered to the tissue, four to six representative images per antrum or corpus were analyzed using FIJI software (National Institutes of Health) to count the number of bacterial spots in each image field.

### Statistical analysis.

Statistical analyses were performed according to the tests specified in each figure legend using Prism v9 software (GraphPad). *P*-values less than or equal to 0.05 were considered statistically significant and are marked with asterisks (*, *P* < 0.05; **, *P* < 0.01; ***, *P* < 0.001).

### Data availability.

The previously published Illumina sequencing reads for the C2-Stock strain (27) are available at the NCBI SRA database (BioProject accession number PRJNA622860). The complete genomes assembled from the PacBio SMRT sequencing performed in this study are available at NCBI GenBank under Bioproject PRJNA786001.
