# Supplementary figures and images for: Helicobacter pylori Chronic Infection Selects for Effective Colonizers of Metaplastic Glands
Source: mBio. 2023 Jan 4;14(1):e03116-22. doi: 10.1128/mbio.03116-22 (PMC9973278; doi:10.1128/mbio.03116-22)

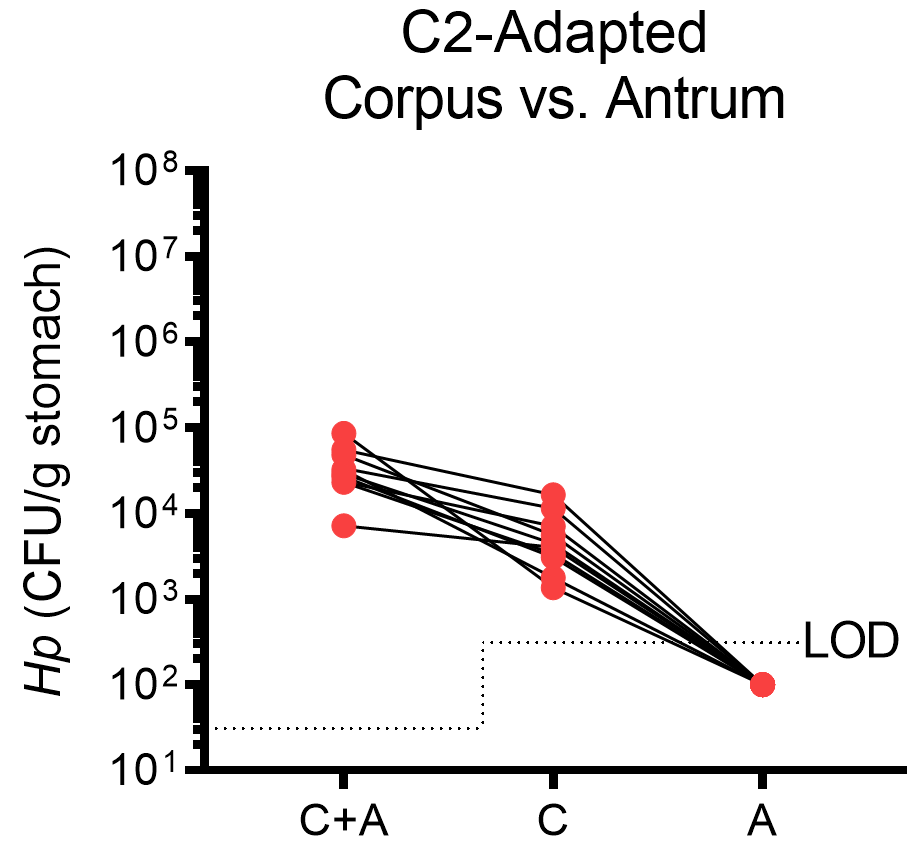

Supplement: FIG S1 [file mbio.03116-22-sf001.tif]

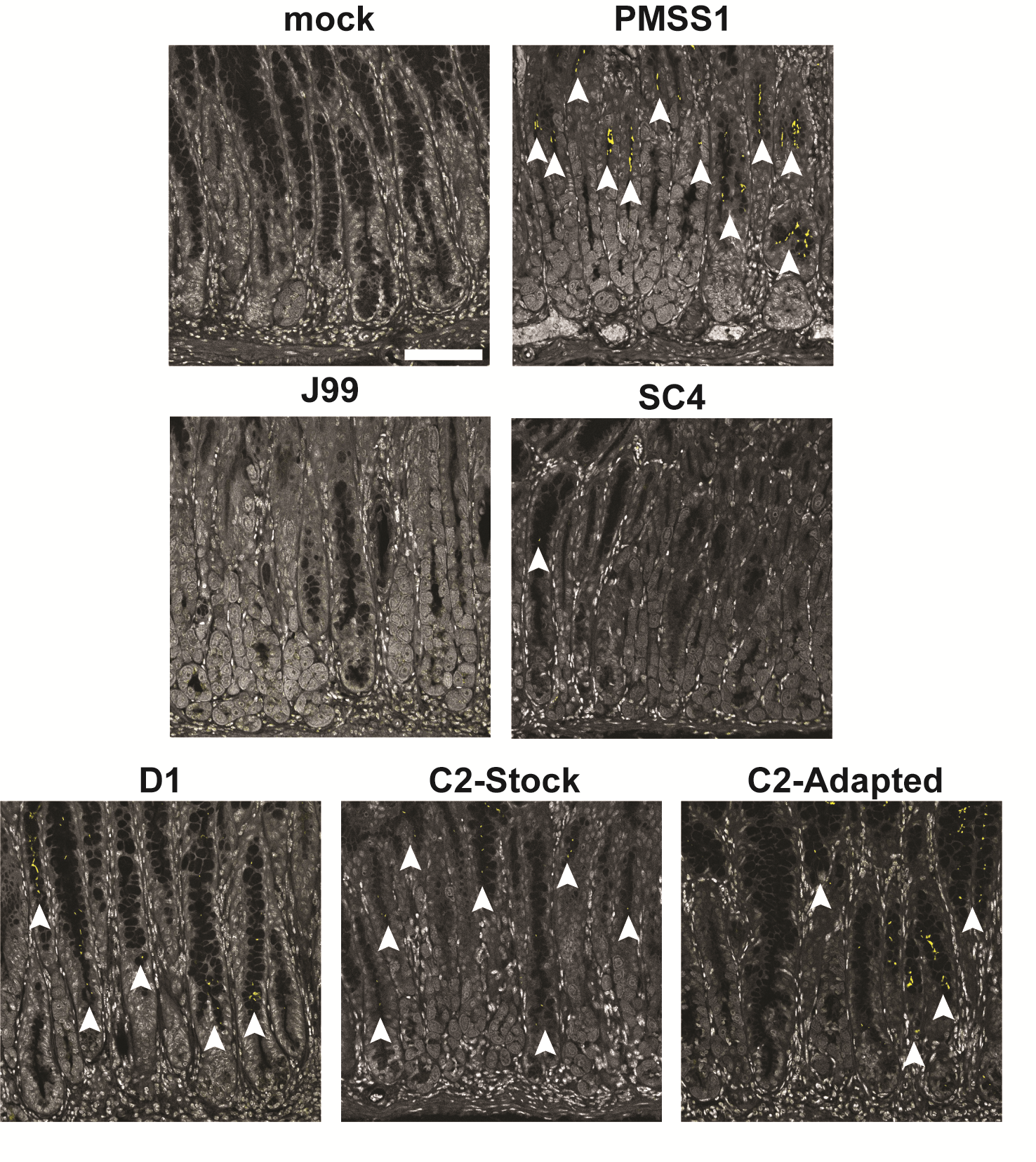

Supplement: FIG S2 [file mbio.03116-22-sf002.tif]

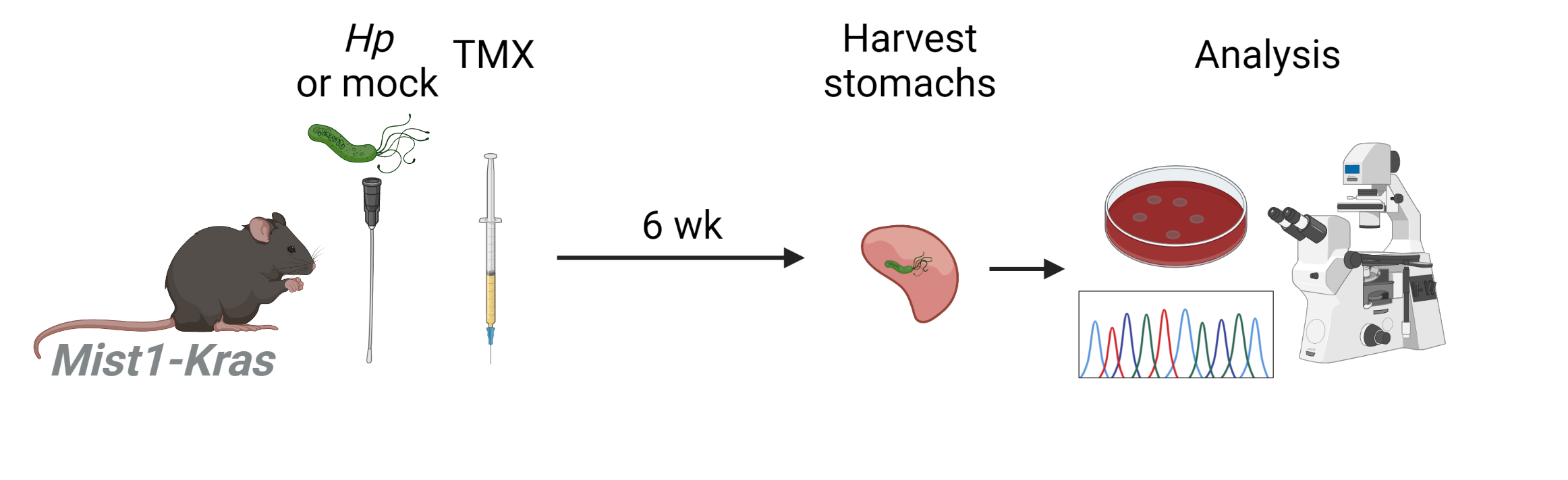

Supplement: FIG S3 [file mbio.03116-22-sf003.tif]

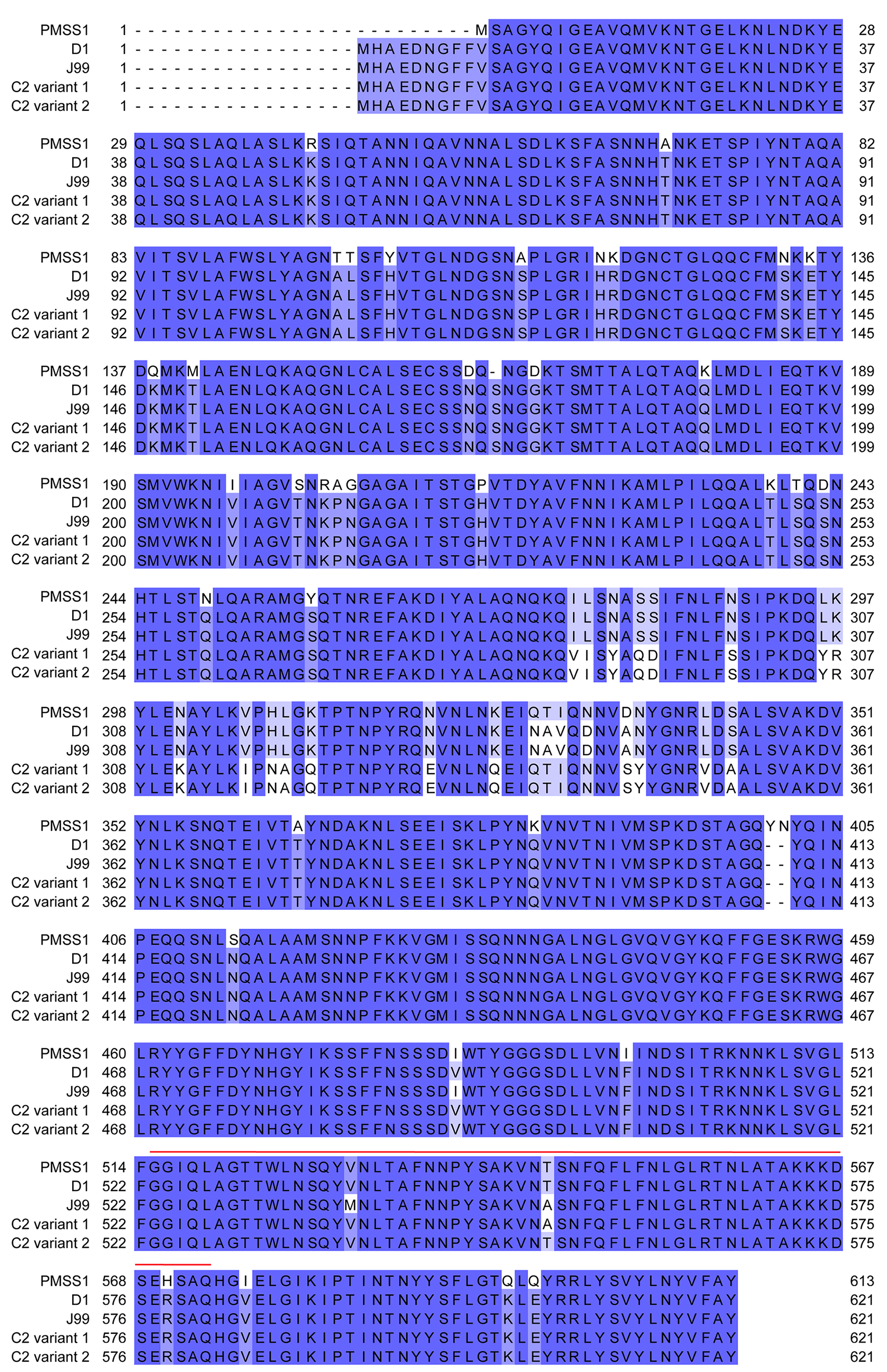

Supplement: FIG S4 [file mbio.03116-22-sf004.tif]
