# Supplementary material for: Helicobacter pylori Chronic Infection Selects for Effective Colonizers of Metaplastic Glands
Source: mBio. 2023 Jan 4;14(1):e03116-22. doi: 10.1128/mbio.03116-22 (PMC9973278; doi:10.1128/mbio.03116-22)
Supplement: TABLE S1 [file mbio.03116-22-st001.pdf]

**Table S1. Nonsynonymous SNPs (n=46) between isolates D1 and C2 stock.**

| Gene ID | Annotation | Description | nSNPs |
|---|---|---|---|
| jhp0034 | *comB8* | DNA transformation competency | 1 |
| jhp0066 | *ureI* | urease accessory protein (urea transporter) | 1 |
| jhp0104 | *fucA* | L-fuculose-1-phosphate aldolase | 1 |
| jhp0116 | *rpl20* | 50S ribosomal protein L20 | 1 |
| jhp0139 | | *unknown* | 1 |
| jhp0151 | *arsS* | signal-transducing protein, histidine kinase | 1 |
| jhp0267 | | *unknown* | 1 |
| jhp0296 | | *unknown* | 1 |
| jhp0314 | *minD* | cell division inhibitor | 1 |
| jhp0325 | *fliF* | flagellar basal-body M-ring protein | 1 |
| jhp0334 | *kgtP* | alpha-ketoglutarate permease | 1 |
| jhp0402 | | *predicted 5'-3' exonuclease* | 1 |
| jhp0429 | *hopJ* | putative outer membrane protein | 1 |
| jhp0468 | | *unknown* | 1 |
| jhp0495 | *cagA* | cag pathogenicity island protein, cytotoxin immunodominant antigen | 2 |
| jhp0554 | *acrB* | acriflavine resistance protein, putative efflux transporter | 1 |
| jhp0575 | *hydB* | quinone-reactive Ni/Fe hydrogenase, large subunit hydrogenase | 1 |
| jhp0620 | *wbpB* | putative lipopolysaccharide biosynthesis protein | 1 |

| | | | |
|---|---|---|---|
| jhp0654 | | predicted tRNA threonylcarbamoyladenosine biosynthesis protein | 1 |
| jhp0728 | *comM* | predicted DNA transformation competence | 1 |
| jhp0785 | *hsdS* | type I restriction enzyme (specificity subunit) | 1 |
| jhp0809 | *katA* | catalase | 1 |
| jhp0899 | *lgt* | prolipoprotein diacylglyceryl transferase | 1 |
| jhp0929 | | plasticity region | 1 |
| jhp0934 | | plasticity region | 2 |
| jhp0935 | | plasticity region | 1 |
| jhp0936 | | plasticity region | 1 |
| jhp0938 | | plasticity region | 1 |
| jhp0956 | | plasticity region | 1 |
| jhp0967 | *metS* | methionyl-tRNA synthetase | 1 |
| jhp0982 | *rps1* | 30S ribosomal protein S1 | 1 |
| jhp1002 | *fucU* | alpha-(1,3)-fucosyltransferase | 3 |
| jhp1065 | *atpF'* | ATP synthase F0, subunit b' ATP synthase B' | 1 |
| jhp1083 | *hopI* | putative outer membrane protein | 1 |
| jhp1121 | *rpoB* | DNA-directed RNA polymerase, beta subunit | 1 |
| jhp1164 | *babB* | outer membrane protein, adhesin | 1 |
| jhp1178 | *pyrE* | orotate phosphoribosyltransferase | 1 |
| jhp1359 | | predicted ABC transport system permease | 1 |
| jhp1371 | *uvrD* | putative DNA helicase II | 1 |
| jhp1392 | | unknown | 1 |

| | | | |
|---|---|---|---|
| jhp1423 | *hsdM* | type I restriction enzyme (modification subunit) | 1 |
| jhp1424 | *hsdR* | type I restriction enzyme (restriction subunit) | 1 |
