# Supplementary material for: Helicobacter pylori Chronic Infection Selects for Effective Colonizers of Metaplastic Glands
Source: mBio. 2023 Jan 4;14(1):e03116-22. doi: 10.1128/mbio.03116-22 (PMC9973278; doi:10.1128/mbio.03116-22)
Supplement: TABLE S2 [file mbio.03116-22-st002.pdf]

Table S2. Insertion or deletion events (n=16) between isolates D1 and C2 stock.

Gene ID	Annotation	Description	Indels
jhp0151	*arsS*	signal-transducing protein, histidine kinase	1
jhp0241		conserved hypothetical secreted protein	1
jhp0451	*pldA*	putative phospholipase A1 precursor (DR-phospholipase A)	1
jhp0709	*amiA*	putative N-acetylmuramoyl-L-alanine amidase	1
jhp0741	*lex2B*	Lipoligosaccharide 5G8 epitope biosynthesis associated protein	1
jhp0888		oxygen-insensitive NAD(P)H nitroreductase	1
jhp0892		unknown	1
jhp0928		plasticity region	1
jhp0934		plasticity region	1
jhp0937		plasticity region	1
jhp0938		plasticity region	1
jhp1002	*fucU*	alpha-(1,3)-fucosyltransferase	1
jhp1171	*csd5*	Cell-shape determining protein	1
jhp1305		unknown	1
jhp1312		unknown	1
jhp1410		type III restriction enzyme (restriction enzyme)	1
