# Supplementary material for: Helicobacter pylori Chronic Infection Selects for Effective Colonizers of Metaplastic Glands
Source: mBio. 2023 Jan 4;14(1):e03116-22. doi: 10.1128/mbio.03116-22 (PMC9973278; doi:10.1128/mbio.03116-22)
Supplement: TABLE S3 [file mbio.03116-22-st003.pdf]

Table S3. Variation between C2 and D1 predicted to be the result of recombination.

Gene ID	Annotation	SNPs	nSNPs	Indels
jhp1300		2	2	3
jhp0659	*sabB*	13	6	0
jhp0662	*sabA*	2	1	1
jhp0857	*hopK*	1	0	0
jhp0870		55	22	3
jhp0649	*homA*	55	18	3
jhp0429	*hopJ*	2	0	0

Genes with alternate stop and start sites

Gene ID	Annotation	Description
jhp1031	jhp1031	alternate stop, truncated protein in isolate D1
jhp0440	jhp0440	alternate stop, truncated protein in isolate D1
jhp1103	*hopQ*	alternate start site, truncated protein in isolate D1
