# Supplementary material for: Helicobacter pylori Chronic Infection Selects for Effective Colonizers of Metaplastic Glands
Source: mBio. 2023 Jan 4;14(1):e03116-22. doi: 10.1128/mbio.03116-22 (PMC9973278; doi:10.1128/mbio.03116-22)
Supplement: TABLE S4 [file mbio.03116-22-st004.pdf]

**Table S4. Antibodies and lectins used to assess *H. pylori* gland colonization and expression of epithelial disease markers.**

| Marker | Species | Dilution | Source | Purpose |
|---|---|---|---|---|
| *Hp* strain PMSS1 | Rabbit | 1:1000 | Manuel Amieva (Stanford University) | Detection of *Hp* |
| Pan-cytokeratin | Mouse | 1:200 or 1:300 | C1801; Sigma (USA) | Identification of gastric epithelium |
| KI-67 | Rabbit | 1:300 | 12202; Cell Signaling Technology (USA) | Proliferating cell marker |
| GS-II | Lectin from *Griffonia simplicifolia* | 1:1000 or 1:2000 | L21415, L21416, L32451; Fisher (USA) | SPEM and mucous neck cell marker |
| CD44v10 (ortholog of human CD44v9) | Rat | 1:25,000 | LKGM002; Cosmo Bio (Japan) | SPEM marker |
| TFF3 | Rabbit | 1:1000 | Daniel K. Podolsky (UT Southwestern) | Intestinal metaplasia marker |
